# Histamine, mast cells and tumour cell proliferation in breast cancer: does preoperative cimetidine administration have an effect?

**DOI:** 10.1054/bjoc.1999.0895

**Published:** 1999-12-08

**Authors:** P F Bowrey, J King, C Magarey, P Schwartz, P Marr, E Bolton, D L Morris

**Affiliations:** UNSW Department of Surgery and Department of Histopathology, The St George Hospital, Kogarah, Sydney NSW 2217, Australia

**Keywords:** histamine, mast cell, proliferation index, Ki-67

## Abstract

Endogenous histamine has been shown to effect growth mechanisms in experimental mammary carcinomas via H2 membrane receptors (Cricco et al, 1994). Both H1 and H2 binding sites are present in human mammary glands but only 75% malignant carcinomas express H2 receptors (Lemos et al, 1995). The presence of mast cells around tumour tissue raises questions concerning the source of histamine in breast tumour tissue. While cimetidine, an H2 antagonist, has been shown to influence the presence of tumour infiltrating lymphocytes (TIL) in colorectal cancer (Adams and Morris, 1994, 1997) that was not found to be the case in breast cancer (Ng et al, 1995). In recent studies tumour cell proliferation, as measured by Ki-67 antibody labelling, has been seen as an additional prognostic indicator in breast cancer (Railo et al, 1993, 1997; Ferno, 1998; Schauer et al, 1998). We investigated the possibility that cimetidine may influence tumour proliferation by blocking the growth-promoting effects of histamine. No relationship between preoperative cimetidine administration and tumour cell proliferation was seen overall. A weak correlation was seen between tissue histamine content and mast cell count which was not influenced by cimetidine. Tumour cell proliferation correlated well with other prognostic indicators such as grade and differentiation. © 2000 Cancer Research Campaign


*British Journal of Cancer* (2000) **82**(1), 167–170
© 2000 Cancer Research Campaign
Article no. bjoc.1999.0895

### Histamine, mast cells and tumour cell proliferation in breast cancer: does preoperative cimetidine administration have an effect?

**PF Bowrey[1], J King[1], C Magarey[1], P Schwartz[1], P Marr[2], E Bolton[1] and DL Morris[1]**

[1]UNSW Department of Surgery and [2]Department of Histopathology, The St George Hospital, Kogarah, Sydney NSW 2217, Australia

**Summary** Endogenous histamine has been shown to effect growth mechanisms in experimental mammary carcinomas via H2 membrane receptors (Cricco et al, 1994). Both H1 and H2 binding sites are present in human mammary glands but only 75% malignant carcinomas express H2 receptors (Lemos et al, 1995). The presence of mast cells around tumour tissue raises questions concerning the source of histamine in breast tumour tissue. While cimetidine, an H2 antagonist, has been shown to influence the presence of tumour infiltrating lymphocytes (TIL) in colorectal cancer (Adams and Morris, 1994, 1997) that was not found to be the case in breast cancer (Ng et al, 1995). In recent studies tumour cell proliferation, as measured by Ki-67 antibody labelling, has been seen as an additional prognostic indicator in breast cancer (Railo et al, 1993, 1997; Ferno, 1998; Schauer et al, 1998). We investigated the possibility that cimetidine may influence tumour proliferation by blocking the growth-promoting effects of histamine. No relationship between preoperative cimetidine administration and tumour cell proliferation was seen overall. A weak correlation was seen between tissue histamine content and mast cell count which was not influenced by cimetidine. Tumour cell proliferation correlated well with other prognostic indicators such as grade and differentiation.
© 2000 Cancer Research Campaign

**Keywords:** histamine; mast cell; proliferation index; Ki-67

The mechanisms of histamine's effect in cancer are probably multi-factorial. Some colon cancer cell lines have been shown to have functional histamine receptors and can be stimulated by local histamine administration (Adams et al, 1994). Histamine also has important effects on immune cells and it was noticed that patients with colorectal cancer receiving pre-resection cimetidine, an H2 antagonist, had a greater chance of having tumour infiltrating lymphocytes (TIL) in their tumours than did the controls (Adams and Morris, 1994, 1997). In contrast, a study by our group found that cimetidine does not influence TIL in breast cancer (Ng et al, 1995). We have reported trends to survival advantage in CR cancer patients treated perioperatively with cimetidine which reached significance in replication error negative tumours (Kelly et al, 1999).

Histamine has been demonstrated to mediate growth control mechanisms in experimental mammary carcinomas, specifically by acting on certain H2 membrane receptors (Cricco et al, 1994), and to play a major role in development and differentiation in the normal rat mammary gland (Davio et al, 1994). Davio et al (1995) found several cell lines derived from mammary gland and human breast carcinomas expressed histamine receptors. In the human mammary gland H1 and H2 binding sites have been demonstrated in both benign and malignant lesions. However, while all benign lesions had both H1 and H2 receptors, only 75% of malignant carcinomas had H2 receptors (Lemos et al, 1995).

*Received 9 February 1999*
*Revised 16 June 1999*
*Accepted 21 June 1999*

*Correspondence to*: DL Morris

A previous study by Reynolds et al (1997) involving some patients in this trial showed the median histamine content of tumour specimens was significantly higher than that of the adjacent healthy tissue. Whether histamine is produced by the tumour cells, mast cells or synthesized elsewhere, the source responsible for the apparent increase in tissue histamine concentration is as yet unknown.

Tumour cell proliferation is a prognostic indicator in breast carcinoma (Tubiana and Courdi, 1989; Railo et al, 1993, 1997; Ferno, 1998, Schauer et al, 1998). The Ki-67 antibody has been recognized for some years as an appropriate antibody to use for demonstrating tumour cell proliferation in breast tumours because it reacts with a nuclear non-histone protein present in all active parts of the cell cycle but absent in G0 (Gerdes et al, 1991; Cattoretti et al, 1992; McCormick et al, 1993). In contrast, proliferating cell nuclear antigen (PCNA), another often used proliferation marker, has a long half-life and may therefore be detected in cells which have recently left the cell cycle or have been involved in DNA repair (Thomas et al, 1993).

Using the Ki-67 antibody proliferation index, this study examines the relationship between tumour-cell proliferation and preoperative cimetidine treatment. It also examines the possible effect of the presence of histamine and mast cells on tumour cell proliferation in breast cancer.

### MATERIALS AND METHODS

### Patient selection

Ethical approval for the study was obtained from the Southern Sydney Area Health Authority. Patients were referred to the trial

Table 1 Histological types of tumour

| Histological type | Cimetidine group | Placebo group |
|---|---|---|
| Ductal | 32 | 32 |
| Lobular | 1 | 3 |
| Tubular | 2 | 1 |
| Mucinous | 0 | 1 |
| Mixed | 1 | 3 |
| DCIS | 1 | 1 |

coordinator (JK) from two surgeons (CM and PS). After providing informed consent to participate in the trial, patients were randomized to receive cimetidine (400 mg twice daily for 5 days prior to surgery) or placebo for the same period. The only exclusion criterion was no other H2 antagonist to be administered for 2 weeks prior to treatment start. Eleven patients in the cimetidine group and nine receiving placebo were on antihypertension medication and, of these, five receiving cimetidine and three receiving placebo were on ACE inhibitors.

### Ki-67 staining and analysis

Immunohistochemistry was performed using a labelled streptavidin–biotin detection system (Dako K0609). Washes were performed between each step in Tris-buffered saline pH 7.6. Four-micrometre sections of paraffin-embedded tissue were mounted on Super Frost Plus slides. The slides were heated at 60°C for 1 h prior to staining. After deparaffinization and rehydration antigenic sites were retrieved by microwaving the sections in Target Retrieval Solution (Dako S1700) for 10 min. Endogenous peroxidase was blocked with 3% hydrogen peroxide in methanol and non-specific adherence of the localization antibody was blocked with 1% skim milk in buffer. For antigen localization sections were incubated in mouse anti-Ki-67 antibody NCL-Ki67-MMI (Novocastra Laboratories, Newcastle, UK) at 1/100 dilution for 1 h at room temperature. The labelled streptavidin–biotin detection system, LSAB+, was used according to the manufacturer's instructions. Antigen sites were visualized with 3,3-diaminobenzidine (DAB) chromogen (Dako S3000). The sections were counterstained with Harris' haematoxylin, dehydrated through increasing concentrations of ethanol, cleared in xylene and coverslipped using DePex mounting medium ready for analysis.

The antigen staining was analysed using Video Pro 32 Image Analyser. Ten high power (400× magnification) representative fields were analysed for each slide providing a proliferation index as a percentage of positive-stained tumour tissue compared to total tumour tissue.

### Mast cell staining and analysis

Four-micrometre paraffin sections were mounted on poly-L-lysine-coated slides. After deparaffinization and rehydration the sections were incubated in a solution of 1% toluidine blue O (CI 52040) in 30% ethanol for 20 min then differentiated with 0.1% acetic acid until the background was almost colourless or pale pink. Sections were then dehydrated, cleared and mounted ready for analysis. A mean count of 6 high power (400× magnification) fields near the tumour margin were taken for each slide analysed.

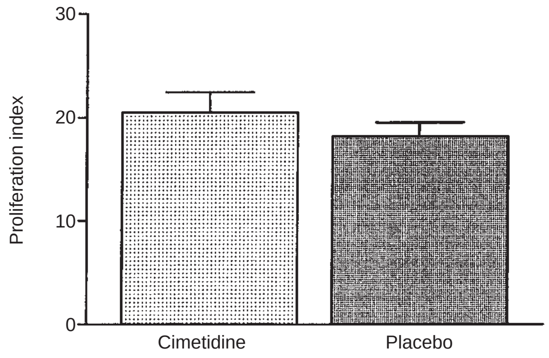

**Figure 1** Comparison of mean proliferation index, measured by Ki-67 labelling, between patients receiving preoperative cimetidine treatment and those receiving placebo

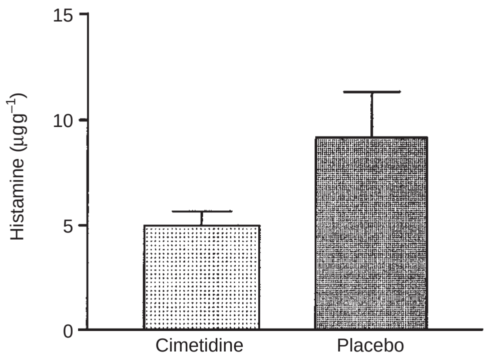

**Figure 2** Comparison of tumour histamine content between patients receiving preoperative cimetidine ($n$ = 9) and those receiving placebo ($n$ = 12)

### Statistical analysis

Mann–Whitney *t*-test for non-parametric data and Pearson's correlation using Prism statistical package.

### RESULTS

A total of 81 patients were enrolled in the trial. Of these, 39 received preoperative cimetidine while 42 received placebo. The age range for patients receiving cimetidine was 31–91 (mean 58) and placebo 32–83 (mean 59). The histological type of tumour is shown in Table 1.

### Comparisons between cimetidine and control group

No significant difference was found between the tumour cell proliferation of patients receiving cimetidine treatment and placebo (Figure 1). The method, materials and results of the histamine assay are published in the paper by Reynolds et al (1998). There appeared to be some difference in tumour tissue histamine content between the two groups with that of the group on placebo being higher than the cimetidine group (Figure 2); however, the difference was not statistically significant ($P$ = 0.1206). When tumour proliferation was compared with tumour histological grade, size, differentiation and lymph node involvement no significant differences were found between the two groups.

© 2000 Cancer Research Campaign

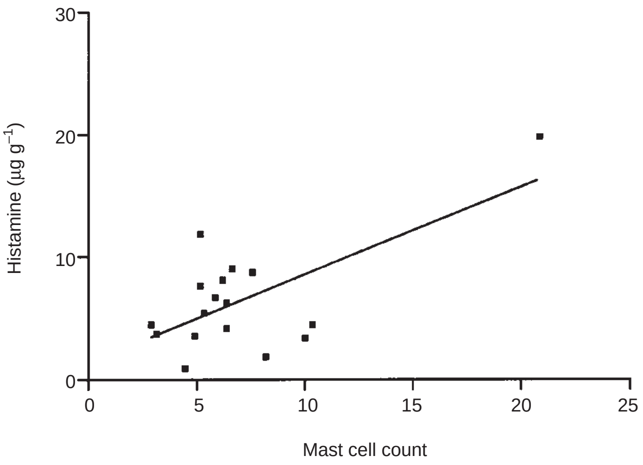

**Figure 3** Correlation between tumour tissue histamine content and mast cell count (*n* = 17)

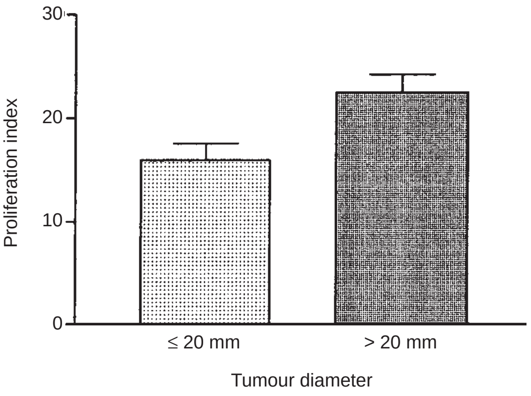

**Figure 4** Comparison of the mean proliferation index for tumours with a diameter of 20 mm or less and tumours with a diameter greater than 20 mm

### General analysis

Mast cell counts were done for patients in which tumour tissue histamine content data was available (Reynolds et al, 1998) and it was found that tumour histamine content correlated positively with mast cell count ($r2 = 0.4411$, $P = 0.0035$) (Figure 3).

There was strong positive correlation between proliferation and grade ($P < 0.01$, $r2 = 0.957$), between proliferation and mitotic score ($P < 0.01$, $r2 = 0.95$) and between proliferation and tumour differentiation ($r2 = 1.0$). There was no correlation between proliferation and lymph node involvement ($P = 0.5416$) or tumour cell histamine content (data not shown). While there was no correlation between proliferation and tumour size overall, when size was divided into quartiles, tumours with a diameter > 20 mm had higher proliferation index than those with diameter < 20 mm (*t*-test $P < 0.0001$) (Figure 4).

### DISCUSSION

We have found no difference in proliferation index between control and cimetidine-treated patients which excluded at least a large direct effect of cimetidine on cellular proliferation in human breast cancer. There are no previous reports of the effect of cimetidine on the proliferation index of human breast cancer cells. Although not significant, grade 1 tumours showed a higher proliferation index in patients on placebo than cimetidine; however, only a small number of patients in the trial had grade 1 tumours (four cimetidine and five placebo). Statistical differences could not be seen between proliferation and any of the variables of size, differentiation and lymph node involvement.

Endogenous histamine is implicated in moderating the growth of experimental mammary carcinomas, treatment with H2 antagonists significantly inhibiting tumour growth and proliferation (Cricco et al, 1994). Some difference in tumour histamine was apparent, being generally greater in the patients on placebo although this did not reach statistical significance. However, the dose of cimetidine being administered achieves a serum concentration of $10^{-6}$ M within 15 min, which persists for 6 h and has the potential to reverse the adverse affects of histamine locally (Adams and Morris, 1997). As tumour histamine was only measured in a small proportion of the patients (nine cimetidine and 12 placebo), analysis of a larger sample is required to determine whether preoperative cimetidine affects histamine levels in breast carcinomas.

Looking at the patients in the trial as one group, a weak positive correlation was found between tumour histamine content and mast cell count, suggesting that more of the tumour histamine present is produced by mast cells than tumour cells. While there is evidence that mast cells are prognostic both in colorectal and breast cancer (Bouzubar et al, 1989; Leonardi et al, 1992). Lemos et al (1995) found that only 75% of breast carcinomas express H2 receptors. Consequently, while mast cells may play a role in breast tumour growth as suggested by Aatomaa et al (1993), the absence of H2 receptors in 25% of breast carcinomas limits the effect of H2 antagonists on tumour growth.

The present study suggests mast cells significantly contribute to the tumour tissue histamine content in breast carcinomas. The tendency towards lower tumour histamine content in patients treated with preoperative cimetidine indicates cimetidine may have an influence on histamine production or mast cell activity.

The role of mast cells in tumour proliferation has been studied mainly in relation to tumour angiogenesis (Roche, 1985*a*, 1985*b*) or connective tissue matrix lysis (Dabbous et al, 1986, 1991). Mast cell histamine has received less attention. Woolley et al (1993) found mast cell products, but not exogenous histamine, increased proliferation in the breast carcinoma cell line 8701-BC. However, this cell line does not express H2 receptors.

Positive correlations between tumour cell proliferation and tumour histological grade and mitotic score have been reported by Bouzubar et al (1989) and Leonardi et al (1992). Tubiana and Courdi (1989) noted that in breast tumours proliferation was significant in relation to prognosis. More recently, proliferation as demonstrated by Ki-67 labelling has been found to be a useful prognostic indicator in breast carcinoma being positively correlated with histological grading (Railo et al, 1993, 1997; Ferno, 1998; Schauer et al, 1998) as seen in the present study. While Viehl et al (1990) found significant correlation between Ki-67 index and mitotic score, unlike our results and those of Bouzubar et al (1989) and Veronese and Gambarcorta (1990), they also found positive correlation with lymph node involvement.

No correlation was seen with proliferation when size was divided into quartiles in our study but tumours with diameter ≤ 2 cm had a significantly lower proliferation index than those > 2 cm ($P < 0.0001$). Veronese and Gambacorta (1990) also found a statistically significant relationship between Ki-67 and tumour size

*© 2000 Cancer Research Campaign*

while Bouzubar et al (1989) who looked at three ranges of size did not. So relationship with size depends on how it is viewed and is only significant as the two extremes are compared.

In conclusion, this study excludes a large effect of a short-term preoperative course of cimetidine on Ki-67 proliferation index in human breast cancer but reports a clear relationship between tumour histamine level and mast cell number. To the authors' knowledge this relationship has not been previously reported.

### REFERENCES

Aaltomaa S, Lipponen P, Papinaho S and Kosma V-M (1993) Mast cells in breast cancer. *Anticancer Res* **13**: 785–788

Adams WJ and Morris DL (1997) Pilot study – cimetidine enhances lymphocyte infiltration of human colorectal carcinoma. *Cancer* **80**: 15–21

Adams WJ, Lawson JA and Morris DL (1994) Cimetidine inhibits in vivo growth of human colon cancer and reverses histamine stimulated in vitro and in vivo growth. *Gut* **35**: 1632–1636

Bouzubar N, Walker KJ, Griffiths K, Ellis IO, Elston CW, Robertson JFR, Blamey RW and Nicholson RI (1989) Ki67 immunostaining in primary breast cancer: pathological and clinical associations. *Br J Cancer* **59**: 943–947

Cattoretti G, Becker MHG, Key G, Duchrow M, Schluter C, Galle J and Gerdes J (1992) Monoclonal antibodies against recombinant parts of the Ki-67 antigen (MIB 1 and MIB 3) detect proliferating cells in microwave-processed formalin-fixed paraffin sections. *J Pathol* **168**: 357–363

Cricco GP, Davio CA, Martin G, Engel N, Fitzsimons CP, Bergoc RM and Rivera ES (1994) Histamine as an autocrine growth factor in experimental mammary carcinomas. *Agents Actions* **43**: 17–20

Davio CA, Cricco GP, Martin G, Fitzsimons CP, Bergoc RM and Rivera ES (1994) Effect of histamine on growth and differentiation of the rat mammary gland. *Agents Actions* **41** Special conference Issue: C115–C117

Davio C, Baldi A, Mladovan A, Cricco G, Fitzsimons C, Bergoc R and Rivera E (1995) Expression of histamine receptors in different cell lines derived from mammary gland and human breast carcinomas. *Inflamm Res* **44**: S70–S71

Ferno M (1998) Prognostic factors in breast cancer: a brief review. *Anticancer Res* **18**: 2167–2172

Gerdes J, Li L, Schlueter C, Duchrow M, Wohlenberg C, Gerlach C, Stahmer I, Kloth S, Brandt E and Flad H-D (1991) Immunobiochemical and molecular biologic characterization of the cell proliferation-associated nuclear antigen that is defined by monoclonal antibody Ki-67. *Am J Pathol* **138**: 867–873

Lemos B, Davio C, Gass H, Gonzalez P, Cricco G, Martin G, Bergoc R and Rivera E (1995) Histamine receptors in human mammary gland, different benign lesions and mammary carcinomas. *Inflamm Res* **44**: S68–S69

Leonardi E, Girlando S, Serio G, Mauri FA, Perrone G, Scampini S, Dalla Palma P and Barbareschi M (1992) PCNA and Ki67 expression in breast carcinomas: correlations with clinical and biological variables. *J Clin Pathol* **45**: 416–419

McCormick D, Yu C, Hobbs C and Hall PA (1993) The relevance of antibody concentration to the immunohistological quantification of cell proliferation-associated antigens. *Histopathology* **22**: 543–547

Ng WK, Vonthethoff LW, Magarey CJ, Adams WJ and Morris DL (1995) The effect of histamine antagonists on the presence of tumour infiltrating lymphocytes in breast cancer: a retrospective study. *Surg Res Comm* **18**: 69–74

Railo M, Nordling S, von Boguslawsky K, Leivonen M Kyllonen L and von Smitten K (1993) Prognostic value of Ki-67 immunolabelling in primary operable breast cancer. *Br J Cancer* **68**: 579–583

Railo M, Lundin J, Haglund C, von Smitten K, von Boguslawsky K and Nordling S (1997) Ki-67, p53, Er-receptors, ploidy and S-phase as prognostic factors in T1 node negative breast cancer. *Acta Oncologica* **36**: 369–374

Reynolds JL, Akhter JA, Magarey CJ, Schwartz P, Adams WJ and Morris DL (1998) Histamine in human breast cancer 8. *Br J Surg* **85**: 538–541

Schauer A, Korabiowska M, Kellner S, Schumacher M, Sauer R, Bojar H and Rauschecker H (1998) Grading system for breast cancer. *Anticancer Res* **18**: 2139–2144

Thomas M, Noguchi M, Kitagawa H, Kinoshita K and Miyazaki I (1993) Poor prognostic value of proliferating cell nuclear antigen labelling index in breast carcinoma. *J Clin Pathol* **46**: 525–528

Tubiana M and Courdi A (1989) Cell proliferation kinetics in human solid tumours: relation to probability of metastatic dissemination and long-term survival. *Radiother Oncol* **15**: 1–18

Veronese SM and Gambacorta M (1991) Detection of Ki-67 proliferation rate in breast cancer: correlation with clinical and pathological features. *Am J Clin Pathol* **95**: 30–34

Vielh P, Chevillard S, Mosseri V, Donatini B and Magdelenat H (1990) Ki67 index and S-phase fraction in human breast carcinomas: comparison and correlations with prognostic factors. *Am J Clin Pathol* **94**: 681–686

*© 2000 Cancer Research Campaign*
